# Fifteen Year Regional Center Experience in Sperm Banking for Cancer Patients: Use and Reproductive Outcomes in Survivors

**DOI:** 10.3390/cancers13010116

**Published:** 2021-01-01

**Authors:** Sara Stigliani, Claudia Massarotti, Caterina De Leo, Elena Maccarini, Fausta Sozzi, Angelo Cagnacci, Paola Anserini, Paola Scaruffi

**Affiliations:** 1 Physiopathology of Human Reproduction, Istituto di Ricerca e Cura a Carattere Scientifico (IRCCS) Ospedale Policlinico San Martino, 16132 Genova, Italy; sara.stigliani@hsanmartino.it (S.S.); elena.maccarini@hsanmartino.it (E.M.); fausta.sozzi@hsanmartino.it (F.S.); paola.anserini@hsanmartino.it (P.A.); 2 Academic Unit of Obstetrics and Gynecology, Department of Neurosciences, Rehabilitation, Ophthalmology, Genetics, Maternal and Child Health (DINOGMI), University of Genova, 16132 Genova, Italy; claudia.massarotti@gmail.com (C.M.); mauricat@unige.it (C.D.L.); angelo.cagnacci@unige.it (A.C.)

**Keywords:** cancer, fertility preservation, sperm cryopreservation, assisted reproduction treatment (ART), ICSI, pregnancy outcome, pregnancy after cancer

## Abstract

**Simple Summary:**

Sperm cryopreservation before gonadotoxic iatrogenic treatments is the only method currently available to preserve fertility in men with cancer. The aims of this study were to report our 15 years of experience, the clinical outcomes of assisted reproductive treatments as well as neonatal characteristics of babies born. We retrospectively reviewed 682 oncological patients who were referred to our center from 2004 to 2019 for fertility preservation. Data regarding cancer diagnosis, age, and the use of frozen semen were analyzed. The cumulative live-birth delivery rate per couple was 35%. No stillbirths, as well as no malformations in the babies born, were recorded. These successful findings demonstrated that pregnancy could be safely achieved using frozen-thawed sperm of cancer survivors who cryopreserved before gonadotoxic therapies.

**Abstract:**

Cancer treatments frequently impair the reproductive ability of patients by damaging spermatogenesis. International guidelines recommend semen cryopreservation to preserve the fertility of oncological adult males and pubertal boys. However, due to the low usage rate of banked samples, not a lot of data on assisted reproductive treatments (ART) success rates in this population and follow-up data for children born are available in the literature. The aims of this study were to report our 15 years of experience, the clinical outcomes of ART as well as neonatal characteristics of babies born. We retrospectively reviewed 682 oncological patients who were referred to our center from 2004 to 2019 for fertility preservation. Over the years, only 26 patients (4%) returned to use their sperm by ART. They were survivors of leukemia and lymphomas (52%), testicular cancer (20%), and other malignant diseases (28%). These couples performed 45 cycles: 34 intracytoplasmic sperm injection (ICSI) plus 11 frozen embryo transfers. A total of 13 children were born, with 35% of the cumulative live-birth delivery rate per couple. No stillbirths or malformations were recorded. These successful findings demonstrated that pregnancy could be safely achieved using frozen-thawed sperm of cancer survivors who cryopreserved before gonadotoxic therapies.

## 1. Introduction

In recent decades, the greater effectiveness of novel oncological treatments has improved the long-term survival of patients with cancer. However, cancer treatments frequently impair the reproductive ability of patients. Among the germ cells, the differentiating spermatogonia in testes are extremely sensitive to the toxic effects of chemotherapeutic agents or localized radiotherapy, regardless of age. On the other hand, Leydig cells are very sensitive to radiation prior to the onset of puberty, whereas in adulthood, they become more resistant [1]. Therefore, adult patients may preserve Leydig cell function and testosterone production following radiotherapy despite being azoospermic.

Therapeutic agents have varying degrees of effects on gametogenesis, depending on sperm quality before treatment, type of malignancy, treatment regimen, and patient susceptibility. Hence, it is not possible to predict reliably whether a patient will become azoospermic or, conversely, whether he will resume partial to normal spermatogenesis after treatment. For this reason, international guidelines recommend that oncologists inform cancer patients of reproductive age about the risk of infertility associated with oncological treatments as well as refer them to specialists in fertility preservation [2,3]. At the present time, cryopreservation of ejaculated semen is the only standard fertility preservation option for oncological adult males and pubertal boys who will be undergoing gonadotoxic treatments [4]. Assisted reproductive treatments (ART), in particular intracytoplasmic sperm injection (ICSI), are required afterward, with pregnancy rates from 12% for intrauterine insemination (IUI) to 32% for ICSI [5,6].

However, there are still open questions on the procedure. According to recent literature, the percentage of patients receiving an offer for sperm cryopreservation, as well as the proportion of patients attempting cryopreservation prior to cancer therapy, vary greatly. Patients with testicular cancer and hematological tumors are most likely to request sperm cryopreservation, followed by bone, soft tissue, and central nervous system tumors. Among hematological tumors, the proportion of lymphoma is higher than others [7,8,9]. The utilization rate of cryopreserved semen is often less than 10%, but the proportion of patients who discard their cryopreserved sperm is also limited to 16%. In addition, there is a positive correlation between the rate of sperm usage and duration of follow-up. Based on these observations, it has been suggested that sperm banking is a long-term program requiring long-term data before definitive conclusions can be drawn, and some authors suggested that the observed usage rate may be an underestimation of the real ultimate usage rate [7,10].

To the best of our knowledge, not a lot of data on ART success rates in this population and follow-up data for children born using frozen-thawed sperm of men with cancer are available in the literature [5,10,11,12,13,14,15,16,17]. Therefore, it is important to keep recording clinical outcomes of cancer survivors and the follow-up of babies born after the use of frozen semen to evaluate the clinical impact of this procedure.

In this study, we performed a retrospective record review of oncological patients at reproductive age who referred to our center from 2004 to 2019 for fertility counseling and preservation before gonatoxic treatments. We longitudinally reported the extent of use of cryopreserved semen, embryological, and clinical outcomes of ART treatments, as well as neonatal characteristics of babies born from these cycles.

## 2. Results

**Figure 1:**
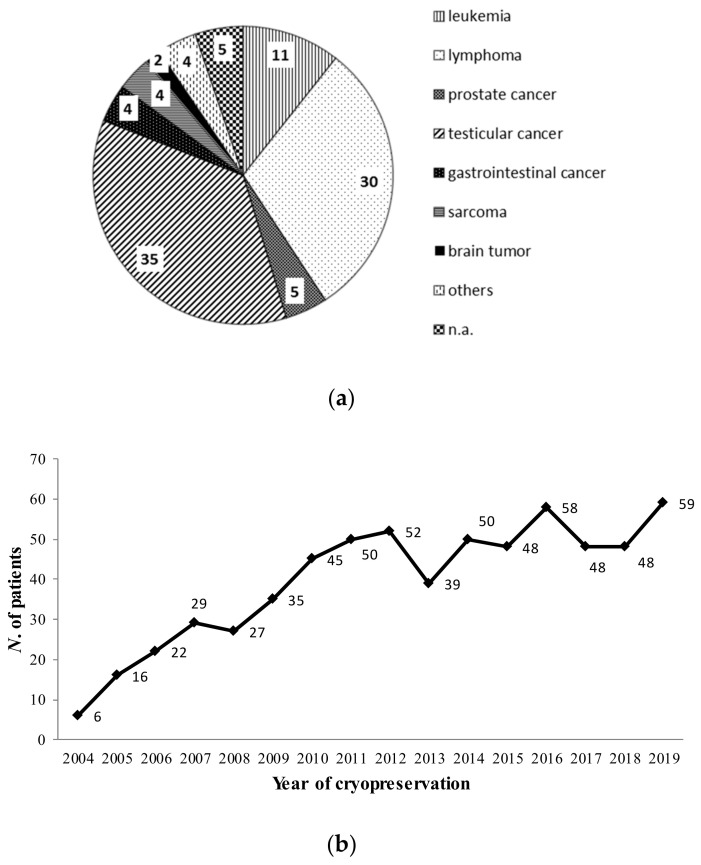
(**a**) Cancer types from patients counseled for fertility preservation at our center. The percentage of each category is represented. n.a.: data not available. (**b**) The number of patients being referred for a successful cryostorage progressively over the years.

A total of 682 patients were referred to our Center. Leukemia and lymphoma (either Hodgkin’s or non-Hodgkin’s) had been diagnosed in 280 (41%) patients, testicular cancer in 242 (35%), and various other types of cancer (i.e., prostate cancers, gastro-intestinal cancers, sarcomas, brain tumors) in 160 (24%) (Figure 1a). Patients’ mean age was 31.6 ± 9.8 years (range 13–65).

Among them, 50 (7%) were not able to produce sperm or they were diagnosed as azoospermic regardless of the oncological disease. They were younger (28.1 ± 8.4 years, range 15–59) than those who stored (*p* = 0.0092). The number of patients being referred for successful storage has increased progressively over the years, as shown in Figure 1b.

### 2.1. Use Rate

Over the years, 32% of patients were lost to follow-up, 46% continued to renew maintenance of their cryopreserved samples at our sperm bank, 10% decided on disposal of their samples, and 2% moved their samples to another center. In 6% of cases, patients died, and the banked samples were discarded. Only 26 patients (4%) returned to use their sperm in order to achieve pregnancy by ART.

**Figure 2:**
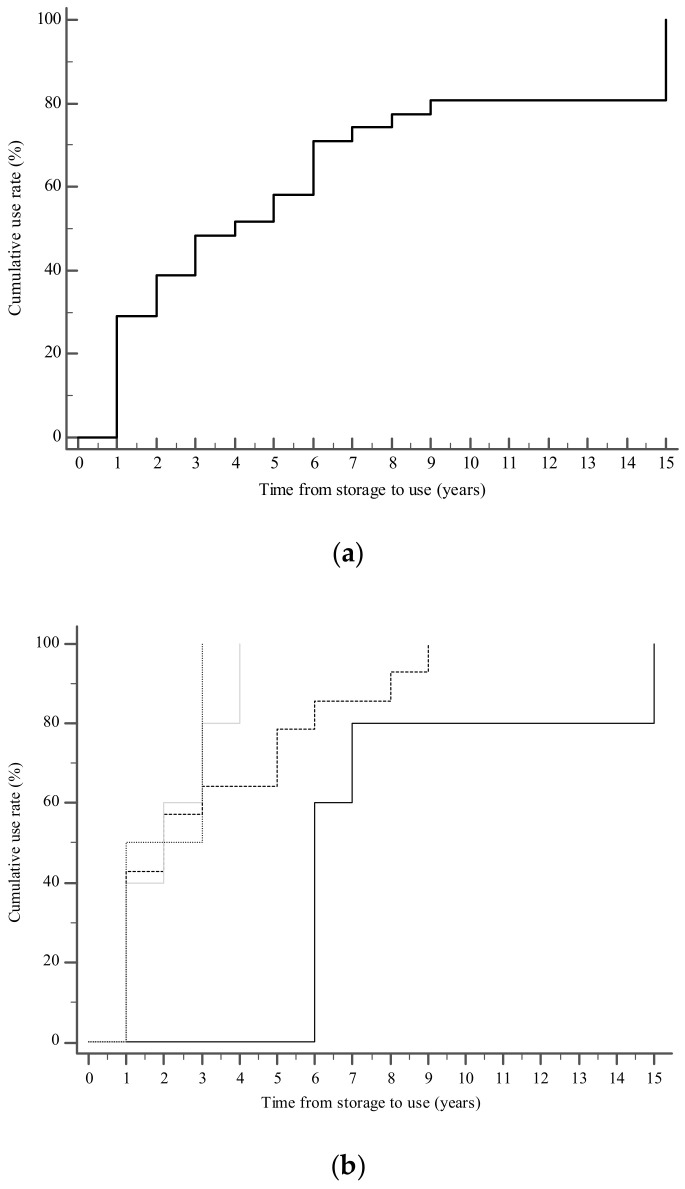
The cumulative rate of first use of frozen semen for the whole cohort (**a**) and according to the age at storage (**b**). The graph was plotted using Kaplan-Meier estimates.

The average time that the samples were cryopreserved in our bank until their first use for ART was 3.3 years. As it is shown in Figure 2a, most of the subjects (22/26, 85%) first thawed their sperm within six years from the time of freezing, with the largest number of them (9/26, 35%) doing so during the first year. There was a decrease in the number of first-time use of sperm over time: from the seventh to the ninth year, only one patient returned each year for the first-time use of his sperm. The longest period of time sperm was cryopreserved before its usage was 15.3 years.

The age of these patients at the time of cryopreservation ranged between 21 and 53 years (average 35.7 ± 7.6 years), and at the time of ART cycle ranged between 26 and 54 years (average 39.0 ± 6.3 years). In comparison to them, men who did not use their frozen sperm and are still banking their material at our center were significantly (*p* = 0.0212) younger at the time of cryopreservation (31.6 ± 9.9 years). In fact, as it is shown in Figure 2b, younger age at the time of sperm banking was associated with a reduced rate of use—starting after six years from storage—with respect to older men.

Men who used their frozen semen for ART were survivors of leukemia and lymphomas (52%), testicular cancer (20%), and other kinds of malignant diseases (28%).

At the time of freezing, sperm concentration (40.0 ± 34.4 million/mL) and total motility (50.4% ± 20.3%) were normal, although the high value of standard deviation signified the marked variability of the ejaculates. A concentration of spermatozoa below the lower reference limit (<15 million/mL) was found in only 27% (7/26) of patients. Among them, 71% (5/7) of cases were men diagnosed with malignant cancers at male genital apparatus (namely, three testicular tumors, one testicular seminoma, and one prostate cancer), one case was affected by chronic myeloid leukemia, and one by Hodgkin’s lymphoma. These patients had significantly fewer spermatozoa per semen sample compared with the other patients (*p* = 0.0043). The lowest mean value of sperm count (0.01 million/mL) was found in a patient with chronic myeloid leukemia. Total sperm motility > 40% was found in 65% (17/26) of patients, and nobody showed sperm motility < 10%. The lowest percentage of motility (10%) was seen in a patient with a testicular tumor and one with testicular seminoma. At post-thaw, a significant reduction of both concentration (10.4 ± 9.8 million/mL) and motility (16.0% ± 16.3%) was observed.

### 2.2. ART Outcomes

The 26 couples of which the male partner thawed his semen performed 45 ART cycles: 34 ICSI plus 11 frozen embryo transfers. Six couples performed two oocyte pick-ups and ICSI cycles, whereas one performed three ICSI after three subsequent oocyte retrievals. The age of females at pick-up was 36.2 ± 4.2 years. Overall, 409 oocytes were retrieved (an average of 11.7 ± 7.2 per cycle), of which 327 were metaphase II (MII) oocytes (9.3 ± 5.4 per cycle). A total of 263 MII oocytes were injected (an average of 7.5 ± 3.8 per cycle), and in three patients, 37 MII oocytes were vitrified. Table 1 summarizes the embryological and clinical outcomes of the 45 ART cycles.

The 34 embryo transfer (ET) of ICSI cycles resulted in 35% clinical pregnancies (12/34, namely 11 after days two to three ET, and one after day five ET), and three additional clinical pregnancies were obtained after the embryo cryopreserved ET, resulting in a cumulative pregnancy rate per couple of 58% (15/26, namely one trigeminal, three twins, and 11 single pregnancies). One singleton was an ectopic pregnancy. The mean age between the females who did or did not achieve a pregnancy was not significantly different (35.9 ± 4.7 vs. 36.9 ± 3.5 years, respectively). No significant correlation was found between pregnancy rate and the storage time (four years for successful cycles and 3.5 for failed ones), the sperm motility after thaw (17% for successful cycles and 15% for failed ones) as well as the post-thaw sperm concentration (9.9 ± 7.9 million/mL for successful cycles and 8.9 ± 7.7 million/mL for failed ones). The lowest total motile sperm count that resulted in a successful pregnancy was 10^4^ in a man diagnosed with a papillary thyroid carcinoma.

A total of 13 children were born (from one tricorionic biamniotic pregnancy, two bicoriotic biamniotic pregnancies, and six singletons), with a 35% (9/26) cumulative live-birth delivery rate per couple. With the exception of one newborn from the transfer of a cryopreserved blastocyst, all the others were from fresh cycles. All pregnancies were uneventful. The perinatal characteristics of the newborns are detailed in Table 2. No stillbirths or malformations were recorded. As detailed in Table 2 all the newborns weighed appropriately for the gestational age (mean: 42° centile, with a mean SDS of −0.17 ± 1.11), with the exception of two pregnancies. One twin pregnancy resulted in the birth via a planned cesarean section of two fetuses at the second and third centile at 37 weeks, and one singleton pregnancy in the birth via urgent cesarean section during preterm labor of a large for gestational age fetus (96° centile).

## 3. Discussion

This study reports on the use rate of cryopreserved semen and pregnancy success rates with the use of thawed semen samples of cancer patients at a semen bank in a university hospital. The Italian Tumor Registry Association (www.registri-tumori.it/cms) reports approximately 196,000 new male cancer patients diagnosed each year in Italy. In young males (0–49 years old), the most frequent tumor is testicular cancer, followed by melanoma and non-Hodgkin’s lymphoma. Consistently, the prevalent diagnoses in patients who were referred to our center were malignant testicular tumors and tumors of lymphatic and hematopoietic tissue. Similar findings were reported by other clinics [5,10,12,13,14,17,18,19].

The majority of our oncological patients presented sperm cells in their ejaculates available for freezing, although men who cryopreserved their semen had a highly variable sperm quality. This may be primarily due to impaired spermatogenesis in cancer patients even before the start of treatments [20,21]. The exact mechanism of this feature is not well understood: it may be associated with the involvement of the immune system [22] and sperm DNA damage because of the malignancy [23]. Similar to other studies [14,24,25], we found the lowest sperm count and motility in men with malignant testis tumors. The correlation between sperm pathology and testicular tumors is most likely associated with altered testicle differentiation during the embryonic development of the gonad [26].

Men who did not produce semen or were azoospermic were younger than those who stored and those who used their samples in ART. Failure of sperm production may be due to emotional distress, taking into account that young men represented the subset of patients highly motivated to cryopreserve their sperm.

The use rate of frozen sperm in our cohort (4%) was comparable with the 3–10% rate experienced by the majority of sperm banks [5,10,11,14,17,19,27,28,29,30]. Based on our data, the rate of usage was the highest within the first year of cryopreservation, and 85% of the patients first returned to claim their sperm within six years. The use of frozen sperm was unsteady and in very small numbers thereafter. Similar findings were shown by Botchan et al. [16] and Kelleher et al. [31]. This behavior could be explained by the fact that immediately after starting anticancer treatments, most patients experience a sharp decrease in their sperm counts, and some even become azoospermic, making cryopreserved sperm their only option to become a father. Moreover, these patients are usually advised by their physicians to avoid conceiving by ejaculated sperm for at least one year from cytotoxic treatments due to the concern for potential chromosomal damage in sperm exposed to cancer therapy [32]. For these reasons, sperm banking should be strongly recommended for all patients with malignant diseases who may wish to have children because no increase in malformation rate has been reported in children conceived with cryopreserved semen of cancer survivors [33], although the available follow-up data are still limited.

Other factors that were not specifically investigated in this study may be related to the “underutilization” of cryobanked sperm, i.e., no need for chemotherapy treatment after surgery, recovery of spermatogenesis, patients’ plans to father long after successfully completed therapy due to uncertainty about long-term prognosis, anxiety regarding potential risks of ART treatment, and fear of transmitting a genetic predisposition to cancer. Financial considerations may also play a role, although, at our center, the costs related to both cryopreservation and infertility treatments are supported by the Public Health System. In the USA and countries where the health care system does not cover these costs, many patients may do not cryopreserve sperm before undergoing cancer treatments because of the high perceived costs of cryopreservation. Actually, sperm cryopreservation prior to undergoing chemo- or radio-therapies remains the most cost-effective strategy for fertility preservation across a range of possible costs associated with surgical sperm retrieval and ART [8].

It is worthwhile noting that the number of patients being referred for sperm cryopreservation has increased progressively over recent years, and in our center, the median years of storage and the median age at the first use for ART were 3.3 and 39 years, respectively. Considering that the age for fatherhood is increasing in developing countries and that patients who were referred to fertility preservation programs were 31.9 ± 9.8 years old, the length of follow-up was probably relatively short, to estimate the real rate of patients who use their frozen samples. In line with this, in our cohort, the age at the time of cryostorage resulted in a relevant factor that determined the usage rate: younger age at the time of sperm banking was associated with a reduced rate of use, suggesting that most of the young patients in our series have not yet decided to father children. These findings have been recently confirmed in a larger series with longer follow-up [34].

Importantly, some couples returned to claim their cryopreserved sperm for many years after the samples have been deposited. In our study, longer storage time did not correlate with semen parameters after thawing nor with lower pregnancy rates. For example, one of our patients—who cryopreserved his semen when he was 24-years old because of a diagnosis of acute myeloid leukemia—claimed his sample for the first time 15 years after completing oncological treatments and fathered one child by means of his thawed sperm. Case reports in the literature described the achievement of pregnancies even after longer periods of semen storage [35].

We are aware of the limitations of our work. First, this paper reports a limited number of pregnancies. However, since in male cancer survivors, the rates of frozen semen usage vary between 3% and 11%, in the literature, there are numerous reviews on male fertility preservation topic, but a paucity of scientific reports about the reproductive outcome of male cancer survivors. Moreover, each study referred to the experience of a single local semen bank and infertility preservation center, thus reporting from two to 33 live-births [34]. That is why it is so important collecting further data about the following effectiveness of ART with spermatozoa frozen before cancer treatments and about the reassuring efficacy of the sperm banking program as the first line fertility preservation option for adult and adolescent male cancer patients. Second, the medical practice has been changed over the 15 years analyzed in the paper. For instance, the mentioned triplet was the result of an ICSI cycle performed in April 2009, when the transfer of three embryos was a possible option applied in ART on the basis of embryo quality. From ten years ago to today, the standard clinical practice in infertility shifted to transferring one or two embryos at most.

In conclusion, we reported the successful live birth of 13 children out of 26 couples who underwent ICSI using sperm frozen for a duration of one to 15 years. Nowadays, pregnancy can be achieved even when cryopreserved semen is the only semen available since micromanipulation techniques can often overcome the poor reproductive potential of frozen-thawed borderline sperm from oncological patients [36]. In this study, we were able to achieve a pregnancy with a sperm count as low as 100,000 sperm/mL by ICSI. Therefore, no minimum sperm count or motility should be required to justify cryopreservation for reproductive-age men before cancer treatment, and an impaired pretreatment semen quality certainly should not rule out sperm banking. In fact, it is broadly believed that for male cancer survivors who undergo ART, ICSI should be chosen over in vitro fertilization (IVF) or IUI in order to reduce the risk of failed fertilization and avoid the exhaustion of a limited sperm stock [6,11,13,17,31]. However, failure to achieve a pregnancy using the cryopreserved semen must be discussed before the semen cryopreservation procedure as well as patients have to be informed that post-thaw semen quality cannot be predicted.

## 4. Materials and Methods

### 4.1. Patients

A total of 682 male cancer patients were referred for fertility counseling to our sperm bank during a period of 15 years (2004–2019) due to scheduled treatments with gonadotoxic potential. All patients were counseled by a specialized biologist from the Andrology Laboratory and fully informed about sperm banking (costs, future possibilities, negative effects of the freezing protocols for the samples, etc.), and afterward, provided written consent before freezing. Among them, 632 (93%) were able to produce sperm cells; the others were diagnosed as azoospermic or were not able to collect sperm.

### 4.2. Semen Collection and Freezing

All samples were obtained by masturbation after three days of sexual abstinence when feasible. All patients cryopreserved sperm immediately after cancer diagnosis and before any treatment. After 10–30 min of liquefaction at 37 °C, semen samples were examined for concentration and motility in a Makler chamber according to the World Health Organization (WHO) guidelines of 1999 [37] and 2010 [38], which did not broadly change during the study period. Each semen sample was diluted 1:1 by dropwise addition of a glycerol-based cryoprotectant with continuous shaking (Freezing medium test yolk buffer; Irvine Scientific, Santa Ana, CA, USA) and then loaded into High-Security sperm straws (Cryo Bio System, L’Aigle, France) that contained 0.5 mL of fluid each. After 10 min incubation at room temperature, the aliquots were incubated for 1 h at +4 °C, gradually cooled by vapor phase nitrogen suspension for 10 min, after which they were transferred into liquid nitrogen containers.

### 4.3. Semen Thawing

Each straw was thawed for 5–10 min at room temperature. Then the semen was added to 1 mL of warmed Sydney IVF Gamete Buffer (Cook Medical, Sydney, Australia) and centrifuged at 1400 rpm for 10 min. The pellet was suspended in 0.5 mL of warmed Sydney IVF Gamete Buffer, and the sample was analyzed for concentration and motility.

### 4.4. Assisted Reproduction Cycles

Standard controlled ovarian stimulation protocols were applied as previously described [39]. Cumulus-oocyte complexes were collected by ultrasound-guided transvaginal follicular aspiration, washed in Sydney IVF Gamete buffer (Cook Medical) and immediately incubated in Sydney IVF Fertilization medium (Cook Medical) at 37 °C in a humidified atmosphere of 6% CO_2_, 5% O_2_, 89% N_2_ using Galaxy 48R incubators (New Brunswick Scientific, Edison, NJ, USA).

After 2 h of incubation, the oocytes were denuded in HEPES-buffered medium (Sydney IVF Gamete medium, Cook Medical) containing 20 IU/mL of Hyaluronidase (Origio, Målov, Denmark). ICSI was performed immediately after denudation according to the conventional procedure. Incubations were performed at 37 °C in a humidified atmosphere of 6% CO_2_, 5% O_2_, 89% N_2_ (Galaxy 48R incubators; New Brunswick Scientific, Edison, NJ, USA). Fertilization was assessed 16–18 h after injection, and embryos with two pronuclei were individually cultured from day one to day three into Sydney IVF Cleavage medium (Cook Medical) and from day three to days five to seven in Sydney IVF Blastocyst medium (Cook Medical).

Day two to three embryos were morphologically scored according to the current consensus system [40]. Arrested embryos were non-viable embryos in which development was arrested for at least 24 h, or in which all the cells degenerated or fragmented.

Standard blastocyst morphological assessment was carried out according to the criteria agreed by an expert panel of scientists [40].

Embryo transfer (ET) was generally performed 72 h after the oocyte collection. However, if the patient had only one to two fertilized oocytes, it could have been done on day two, and if she had at least four good quality embryos on day three, day five transfer at the blastocyst stage was considered.

Surplus embryos that developed up to the blastocyst stage were cryopreserved by vitrification [41], using a commercial kit (Vit Kit-Freeze, Fujifilm Europe BV, Tilburg, The Netherlands). For the warming procedure, the Vit Kit-Thaw (Fujifilm Europe BV) was used, and the blastocysts were cultured at 37 °C for at least 2 h before ET into the same medium used for embryo culture.

### 4.5. Data Analysis

Other than general patients’ demographic and clinical data, we retrieved information for each cycle performed by using cryopreserved semen. We collected data about sperm count and motility before the freezing and after the thawing. The reported outcomes of cycles were fertilization rate (defined as the ratio between the number of fertilized oocytes and the number of MII oocytes injected), cleavage rate (defined as the ratio between the number of cleaved embryos and the number of fertilized oocytes), quality of embryos, blastocyst development rate (defined as the ratio between the total number of blastocysts formed and the number of embryos cultured up to days five to seven), implantation rate (defined as fetal cardiac activities at 12 weeks of gestation divided by the number of transferred embryos), pregnancy rate (defined as pregnancies with at least one gestational sac divided by the number of embryo transfers), miscarriage rate (defined as abortions divided by the number of pregnancies), and birthweights (expressed as a percentile and standard deviation score (SDS) for gestational age, according to the Italian reference curves [42]). Categorical variables were presented as percentages.

Continuous variables were presented as mean ± SD and range. Data were analyzed by Wilcoxon test (paired samples, for sperm parameters before freezing and after thawing) and Mann–Whitney test using MedCalc^®^ software (Mariakerke, Belgium). A *p*-value < 0.05 was considered statistically significant.

## 5. Conclusions

As cancer survival has largely increased during the last decades, infertility is recognized as one of the most devastating long-term side effects of antineoplastic therapies [43]. At this moment, semen cryopreservation is the only established and reliable method to preserve fertility [9,44]. Our 15 years of experience with ICSI in male cancer survivors is a further confirmation that semen can be stored for a reasonable long time without affecting pregnancy rates and that the use of cryopreserved semen leads to successful pregnancies in approximately half of the couples. These findings may represent reassuring information for patients about the potential benefits of sperm banking programs and may encourage them to store and then uptake their semen.

For the above reasons, increased awareness of oncologists, hematologists, urologists, internists to advances in semen cryopreservation procedures and ART is a high priority. At the same time, it is reproductive medicine specialists’ responsibility to inform physicians who interact with young male cancer patients about the possibilities in fertility preservation before gonadotoxic treatments and the expected pregnancy outcomes using frozen semen. The usefulness of referring all newly diagnosed young male cancer patients to sperm banking facilities before cancer treatment can be explained by either describing the possibilities of becoming sterile after the treatment and informing patients of the encouraging ART results.

## Figures and Tables

**Table 1:** Embryological and clinical outcomes of ART (34 ICSI + 11 frozen embryo embryo transfer (ET)).

Parameter	*N*. (%)
Fertilization (%)	180/263 (68)
Cleavage (%)	175/180 (97)
Top quality embryo (%)	89/175 (51)
Blastulation (%) ^1^	27/96 (28)
Delayed ET (freeze-all) (%)	1/35 (3)
Cycles with day 2–3 ET	30
Implantation (%)	15/69 (22)
Pregnancy per cycle (%)	11/30 (37)
Live-birth rate per cycle (%)	11/30 (37)
Miscarriage (%)	3/11 (27)
Ectopic pregnancy (%)	1/11 (9)
Cycles with day 5 ET	4
Implantation per cycle (%)	1/4 (25)
Pregnancy per cycle (%)	1/4 (25)
Live-birth rate per cycle (%)	1/4 (25)
Miscarriage (%)	0
*N*. ET in freeze-thaw cycles	11
Implantation per cycle (%)	4/13 (31)
Pregnancy per cycle (%)	3/11 (27)
Live-birth rate per cycle (%)	1/11 (9)
Miscarriage (%)	2/11 (18)
Cumulative pregnancy rate per cycle (%)	15/45 (33)
Cumulative live-birth rate per cycle (%)	13/45 (29)
Cumulative miscarriage (%)	5/15 (33)
Cumulative live-birth delivery rate per couple (%)	9/26 (35)

^1^ Blastulation rate is expressed as *N*. of developed blastocysts per all embryos that were cultured up to blastocyst-stage. ART: assisted reproductive treatments; ICSI: intracytoplasmic sperm injection.

**Table 2:** Perinatal characteristics of newborns.

Parameter	*N*.
*N*. newborns	13
*N*. lost follow-up	0
*N*. ongoing pregnancies	0
Birthweight (grams) ^1^	
Total	2610.2 ± 643.0
Singletons	3254.0 ± 183.3
Multiples	2075.0 ± 224.5
*N*. birthweight < 2500 g	
Total	7
Singletons	0
Multiples	7
Gestational age (weeks) ^1^	
Total	37.3 ± 1.8
Singletons	38.0 ± 1.4
Multiples	36.0 ± 2.0
*N*. prematurity < 37 weeks	
Total	2
Singletons	0
Twins	2
Birthweight centiles ^1^	
Total	41.7 ± 24.0
Singletons	42.6 ± 23.5
Multiples	30.2 ± 38.5
SDS-score ^1^	
Total	−0.17 ± 1.11
Singletons	0.08 ± 1.18
Multiples	−0.74 ± 0.76

^1^ Values are mean ± SD unless otherwise stated.

## Data Availability

The data presented in this study are available on request from the corresponding author.
